# Areas of Increasing Agricultural Abandonment Overlap the Distribution of Previously Common, Currently Threatened Plant Species

**DOI:** 10.1371/journal.pone.0079978

**Published:** 2013-11-08

**Authors:** Takeshi Osawa, Kazunori Kohyama, Hiromune Mitsuhashi

**Affiliations:** 1 National Institute for Agro-Environmental Sciences, Tsukuba, Japan; 2 The Museum of Nature and Human Activities, Hyogo, Japan; DOE Pacific Northwest National Laboratory, United States of America

## Abstract

Human-driven land-use changes increasingly threaten biodiversity. In agricultural ecosystems, abandonment of former farmlands constitutes a major land-use shift. We examined the relationships between areas in which agriculture has been abandoned and the distribution records of threatened plant species across Japan. We selected 23 plant species that are currently identified as threatened but were previously common in the country as indicators of threatened plant species. The areas of abandoned farmlands within the distribution ranges of the indicator species were significantly larger than the proportion of abandoned farmland area across the whole country. Also, abandoned farmland areas were positively correlated with the occurrence of indicator species. Therefore, sections of agricultural landscape that are increasingly becoming abandoned and the distribution ranges of indicator species overlapped. These results suggest that abandoned farmland areas contain degraded or preferred habitats of threatened plant species. We propose that areas experiencing increased abandonment of farmland can be divided into at least two categories: those that threaten the existence of threatened species and those that provide habitats for these threatened species.

## Introduction

Agriculture changes natural terrestrial ecosystems to terrain managed by human enterprise [1,2]. Although traditional low-impact agriculture maintains biodiversity [3], recently adopted agricultural practices seriously threaten species diversity [4,5] especially marked in traditional agricultural landscapes in temperate zones [6,7,8,9,10]. 

 Changes in agricultural land use follow two trajectories. Tracts of land under traditional farm management have often been either abandoned or subjected to intensive modern agricultural practices [11,12]. Both of these fates can influence biodiversity [12], and both are usually driven by economic pressures [10,12,13,14,15]. Agricultural abandonment is widespread in several regions of the world [16], including North America [17], northern Europe [8], southern Europe [18] and monsoonal Asia (Japan) [10,19]. 

 Several studies have evaluated the specific relationships between agricultural abandonment and regional biodiversity at a local scale in Japan [10,12,14,20,21,22], but the general relationship remains unclear. Agricultural abandonment sometimes negatively influences regional biodiversity [10,12,14,22], although Ikegami et al. [23] demonstrated that abandoned rice fields provide habitat that might harbour threatened plant species. Despite such contradictions, the Japanese National Biodiversity Strategy 2010 classifies increasing agricultural abandonment as an “under-use crisis” that threatens biodiversity. Thus, agriculture in its traditional form is seen as a mechanism maintaining habitats that are hot spots for species diversity; these habitats are threatened by decreased human activity [24]. In actuality, however, the total area of abandoned agricultural lands has approximately doubled over the past 15 years from 217,000 ha in 1990 to 396,000 ha in 2005 [25] (Figure S1). The rate of species extinctions may be increasing with the recent dramatically increased rates of agricultural land abandonment, although this relationship remains moot if the process might also provide habitat for threatened plants [23]. In the present study, we investigated relationships between agricultural abandonment and the distributions of threatened species at large geographical scales across the Japanese archipelago. If we found a general relationship between these plants and agricultural abandonment, we could propose that these issues should be taken into account when drawing up plans for the management of abandoned land previously used for traditional agriculture. To the best of our knowledge, this concept has not been incorporated into any previous studies on abandonment of landscape units with this history of shifting land use.

 Japan has many threatened plant species [26]. From these, we selected several that were formerly distributed over broad regions of the country, but are now declining across their previous ranges. We used these previously common but currently threatened species (hereafter, proposed threatened, or PT, species) as indicators of biological diversity. We selected such taxa because the factor(s) responsible for the declines is likely common to wide areas of former distribution ranges. The rural flatlands of most of Japan have a long history of agriculture, and Japanese farmland areas were mainly established with “satoyama”, which is the traditional Japanese rural landscape consisting of farming and forestry villages, and the semi-natural environments that surround them [27]. Thus, to some extent, the PT species are likely to depend on farmland areas for habitat.

 To investigate the relationships between the geographical pattern of agricultural abandonment and the distributions of PT species in Japan, we examined agricultural statistical data assembled by the government as well as a governmental, countrywide, public database for the distribution records of PT species (see Materials and methods). We addressed the following question: Does a relationship exist between currently threatened plant species and areas abandoned from agriculture in Japan?

## Materials and Methods

### Study area and analysing unit

**Figure 1:**
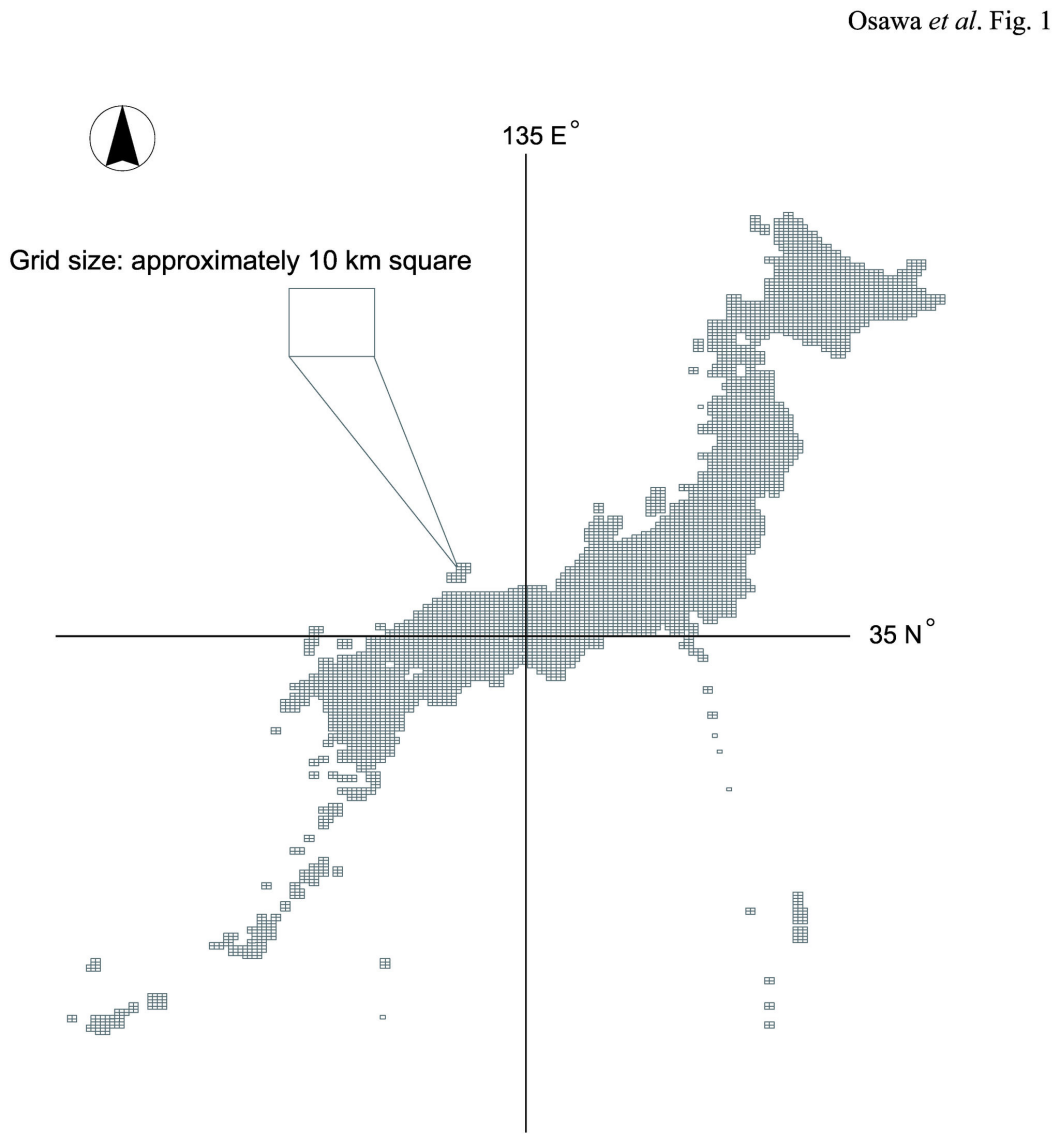
Study area and units selected for this study. Locations and sizes of grids were defined by the Japanese government.

The study was conducted across all of Japan (Figure 1). A grid size of approximately 10 km was used for the analyses. This is the Japanese Standard Second Mesh (hereafter, 2nd mesh), which corresponds to 1:25 000 map grids (Figure 1). The locations of the 2nd mesh grids were determined arbitrarily by the Japanese government for comparing several statistics, including population, infrastructure and prevention of disasters at this scale of geographical resolution [28]. We obtained 2nd mesh information as polygon data from the Japan Integrated Biodiversity Information System available at the Biodiversity Centre of Japan [29]. In the past 15 years, the rate of agricultural abandonment in Japan has approximately doubled [25]. This is a critical issue in Japanese agricultural planning, not only for biodiversity but also for food production [25].

### PT species selection and occurrence records

In Japan, Japanese Prefectural Red Data Books (PRDBs) are created by local authorities in all 47 prefectures in the country. Listed taxa occur locally and are subject to decline within the prefecture, which is determined using prefecture-specific criteria. Using all of the PRDBs, it is possible to identify species that had formerly broad distributions but have declining populations, namely PT species, across wide areas of Japan. Species with limited habitat and/or small distribution ranges, such as specific islands or high montane areas, were ruled out of consideration for our purposes. We defined the PT species, as listed in the PRDBs, for at least 35 prefectures that occupy 75% of the national surface area. To assign PT status to species, we collected the 2010 PRDBs from all 47 prefectures (Table S1). Subsequently, we listed all plant species records from PRDBs and counted the numbers of each. In this procedure, the threatened category assignment in each PRDB was ignored because the prefectural definitions were variable. 

 Distribution records of PT species were obtained from J-IBIS [29]. We used occurrence records of threatened plants in 2nd meshes in J-IBIS that were compiled from Japanese National Red Lists (NRLs) in 2001 and 2007. These data were corrected using observations and information from regional experts [30]. Distribution records were refined by conducting additional surveys over a 5-year period based on previous records. Thus, these data are cumulative occurrence records from past to present, include extinctions and cover all of Japan. We took PT species records from this data set and selected those with at least 50 records. Thus, we obtained distribution records for 23 PT plant species per 2nd mesh grid unit for the whole of Japan (Table 1). These records were used in our analysis.

**Table 1:** The statuses of the 23 PT species selected.

Family	Name	Local RDB	National RL rank	Main habitat	Life forms
Azollaceae	*Azolla japonica*	35	VU	Paddy, Ditch	perennial fern
Hydrocharitaceae	*Blyxa echinosperma*	42	VU	Paddy, Ditch	annual
Alismataceae	*Caldesia parnassifolia*	37	VU	Pond, Lake, Paddy	perennial
Compositae	*Gnaphalium hypoleucum*	40	VU	Forest edge	annual
Isoetaceae	*Isoetes japonica*	36	NT	Pond, Paddy, Ditch	perennial fern
Compositae	*Ixeris chinensis subsp. strigosa*	40	VU	Grassland, Roadside	perennial
Labiatae	*Leonurus macranthus*	39	VU	Grassland	perennial
Marsileaceae	*Marsilea quadrifolia*	46	VU	Paddy, Ditch	perennial fern
Pontederiaceae	*Monochoria korsakowii*	39	NT	Lake, River, Ditch, Paddy(fallow)	annual
Labiatae	*Mosla japonica*	36	NT	Roadside	annual
Najadaceae	*Najas gracillima*	40	NT	Pond, Paddy	annual
Menyanthaceae	*Nymphoides peltata*	40	NT	Lake, Pond, Paddy	perennial
Saxifragaceae	*Penthorum chinense*	41	NT	Swamp	perennial
Polygonaceae	*Persicaria foliosa var. paludicola*	36	VU	Swamp, Paddy(fallow), Pond	annual
Polygonaceae	*Persicaria taquetii*	36	VU	Swamp, Paddy	annual
Potamogetonaceae	*Potamogeton berchtoldii*	44	NT	Lake, Pond, River, Ditch	perennial
Salviniaceae	*Salvinia natans*	40	NT	Paddy, Ditch	annual fern
Compositae	*Saussurea pulchella*	39	VU	Grassland, Forest edge	annual
Sparganiaceae	*Sparganium fallax*	41	NT	Lake, Pond, River	perennial
Sparganiaceae	*Sparganium stenophyllum*	41	VU	Lake, Pond	perennial
Trapaceae	*Trapa incisa*	37	VU	Lake, Pond, Ditch, Paddy	annual
Lentibulariaceae	*Utricularia uliginosa*	43	NT	Swamp	perennial
Asclepiadaceae	*Vincetoxicum pycnostelma*	44	NT	Grassland	perennial

For the National Red List (NRL) ranks, VU = vulnerable and NT = near-threatened according to the NRL for Japan. Primary habitat and life-form were derived from the NRL and books of endangered plants.

Local RDBs indicated the number of prefectures that assigned the species threatened status.

In addition to the PRDBs, a NRL exists, and species therein have well-known ecological traits, distributions and habitat requirements because conservation research and/or activities have tended to focus on PRDBs and/or NRL-listed taxa [31,32]. Indeed, the NRL publication itself contains considerable relevant information about each of the species [26]. For each PT species, we used the primary habitat and the threatened category assignment in the NRL as reference information. We referred to both the current NRL [26] and the book of endangered plants by Yahara and Nagata [33].

### Agricultural land-use data set

We obtained agricultural land-use data from the Census for Agriculture, Forestry and Fisheries (CAFF) data set [34]. This census is conducted every 5 years by the ministry. We used data released in 2005, which matched years when PT species records were released (2001 and 2007). 

The CAFF data set includes statistics on agricultural land use, e.g. total farmland and breakdown of farmland into categories such as abandoned areas and pasture [34]. These records were summarised using old municipality units. The average area of a municipality unit was 148.32 km^2^ [35]. To convert the municipality units into 2nd mesh units, we divided the municipality units into 100-m (i.e. 1 ha) mesh units, and reconstructed the 2nd mesh. The 2nd meshes could contain 10 000 m^2^ that was divided into 100-m meshes. First, we divided the municipality units into 100-m mesh units and partitioned both total farmland and agriculturally abandoned areas equally. If one of these mesh units included two or more municipality units, we assigned the mesh unit to the dominant municipality; i.e. we assigned the mesh to the single municipality that represented the largest portion of the mesh area. Thus, we assumed that the proportions of total farmland and abandoned areas were equally distributed in the 100-m grid within the same municipality. Subsequently, we reconstructed 2nd mesh units using 100 × 100-m (i.e. 10 000 m^2^) meshes and summed area values for both total farmland and abandoned areas. We ignored the numerical error that might occur by dividing the municipality units into the 100-m mesh because the 2nd mesh size was sufficiently larger than the 100-m mesh. We used 100-m small mesh units rather than 1-km grids as the minimum area for reconstructing the 2nd mesh because the smallest municipality unit was 1.27 km^2^ [34,35], and the 1-km mesh did not meet our accuracy requirement. We reconstructed 2nd meshes and calculated the total farmland and abandonment area in each 2nd mesh unit. 

### Analysing relationships between PT species records and areas of farmland

The 23 PT species records per 2nd mesh unit were merged with the data for both total farmland area and abandonment area coverage in the same units. We used Mann–Whitney *U*-tests to compare average abandoned area between the ranges of PT species’ distributions and all of Japan per 2nd mesh unit for both 2001 and 2007. Mann–Whitney *U*-tests are robust for relatively small samples [36] such as the PT occurrences in this study. We did not use total farmland area in this analysis. In this procedure, we excluded 2nd meshes that had no abandoned area per 2nd mesh unit. If species occurrences overlapped areas where abandonment is increasing, the average abandoned area within the PT species’ distribution would be significantly larger than the abandoned area across the whole country. 

We also used generalised linear models (GLMs) with logistic error distribution (log-link) and Wald tests to examine the effects of abandoned area and total farmland area for each PT species occurrence. We used total farmland as an explanatory variable in GLM analyses to determine if PT species depend on farmland and to separate the effects of abandoned areas and farmland. The correlation value between abandoned area and total farmland was 0.0553. We deemed these results “meshes”, which have PT species records as spatial presence units. The other results did not have PT species records as absence units to apply to the GLMs. Although our absence units include pseudo-absences, which means that the species was not detected but does actually occur, we found this analysis sufficient to provide general trend information on the preparation of farmland type requirements for each PT species. 

Additionally, we conducted a “bagging GLM” analysis, which combines GLM and bootstrap aggregation, a machine learning technique [32]. The bagging GLM model creates a large number of GLM models using the same composition of presence/absence data derived from all data and then combines these models. This method has high predictive ability with presence data and also has advantages when analysing presence-only records for broad range records, such as the data used in this study [32]. A bagging GLM cannot serve as a significance test but can show coefficients in each explanatory variables [32]. In this study, we randomly selected a fixed number of supposed absence data from all meshes without PT species records as presence data, repeated the process to create 5 000 GLM models, and then combined these models into a final model. The final model was used as a linear predictor based on the average values of the coefficients of the 90% confidence intervals of all 5 000 models [32]. We used the statistical package R ver. 2.14 [37] for all analyses.

## Results

Among 23 PT species, *Potamogeton berchtoldii* and *Vincetoxicum pycnostelma* were assigned to the endangered species category by 44 prefectures (Table 1), the highest number among PT species considered for analysis. *Vincetoxicum pycnostelma* had the highest number of occurrence records in both data sets (Table 2). Three Compositae were among the selected PT species (Table 1). Of the 23 PT species, 12 grew in paddy fields, 9 in ponds and 8 in ditch habitats (Table 1). Forest edge and roadside habitats each contained two of the selected species (Table 1). According to the current NRL [26], 12 species were classified as vulnerable (VU), and 11 species were near threatened (NT) (Table 1). 

**Table 2:** Results of *Mann-Whitney U-tests* comparing the proportional area of abandoned farmland within the distribution ranges of PT species and the proportional area of abandoned farmland across the whole of Japan in 2001 and 2007.

Species name	Number of occurrences in 2001 mesh records	Average abandonment area with the species records (Mean ± S.D.)	p	Number of occurrences in 2007 mesh records	Average abandonment area with the species records (Mean ± S.D.)	p
All of Japan released at 2005 data set	4854	80.62 ± 115.39	(Whole Japan)			
		87.88 ± 117.71	(Excluded no-abandonment meshes)			
*Vincetoxicum pycnostelma*	253	141.86±132.29	***	357	155.41±148.93	***
*Penthorum chinense*	226	209.72±191.95	***	288	205.75±182.59	***
*Utricularia uliginosa*	210	124.5±107.55	***	256	121.93±110.76	***
*Isoetes japonica*	182	184.3±157.9	***	255	176.62±157.71	***
*Salvinia natans*	163	200.32±167.53	***	247	186.77±156.2	***
*Leonurus macranthus*	140	130.69±103.29	***	172	130.66±105.41	***
*Potamogeton berchtoldii*	121	154.5±144.18	***	202	154.29±134.08	***
*Azolla japonica*	118	219.14±210.19	***	152	209.07±192.68	***
*Gnaphalium hypoleucum*	117	184.76±166.72	***	136	132.03±123.93	***
*Sparganium stenophyllum*	104	140.75±140.18	***	159	139.76±140.04	***
*Marsilea quadrifolia*	102	218.26±211.95	***	141	205.45±205.8	***
*Saussurea pulchella*	101	122.31±99.39	***	118	118.6±97.4	***
*Sparganium fallax*	98	151.05±127.61	***	141	148.5±127.04	***
*Monochoria korsakowii*	85	179.9±157.96	***	120	161.66±155.29	***
*Blyxa echinosperma*	83	155.01±122.86	***	113	172.48±144.82	***
*Mosla japonica*	82	155.73±141.39	***	99	161.33±138.33	***
*Trapa incisa*	71	159.74±122.92	***	81	157.13±129.99	***
*Ixeris chinensis subsp. strigosa*	71	159.75±132.43	***	93	158.71±125.53	***
*Nymphoides peltata*	65	206.01±194.63	***	88	196.58±224.01	***
*Persicaria taquetii*	62	161.82±154.97	***	81	154.77±157.51	***
*Caldesia parnassifolia*	56	148.22±148.38	***	73	150.31±140.7	***
*Persicaria foliosa var. paludicola*	54	231.59±201.91	***	85	218.87±201.06	***
*Najas gracillima*	52	186.86±135.56	***	108	191.96±165.44	***

Significant effects identified by U-tests are provided (n.s.: not significant, *: p<0.05, ** p<0.01, *** p<0.001)

**Figure 2:**
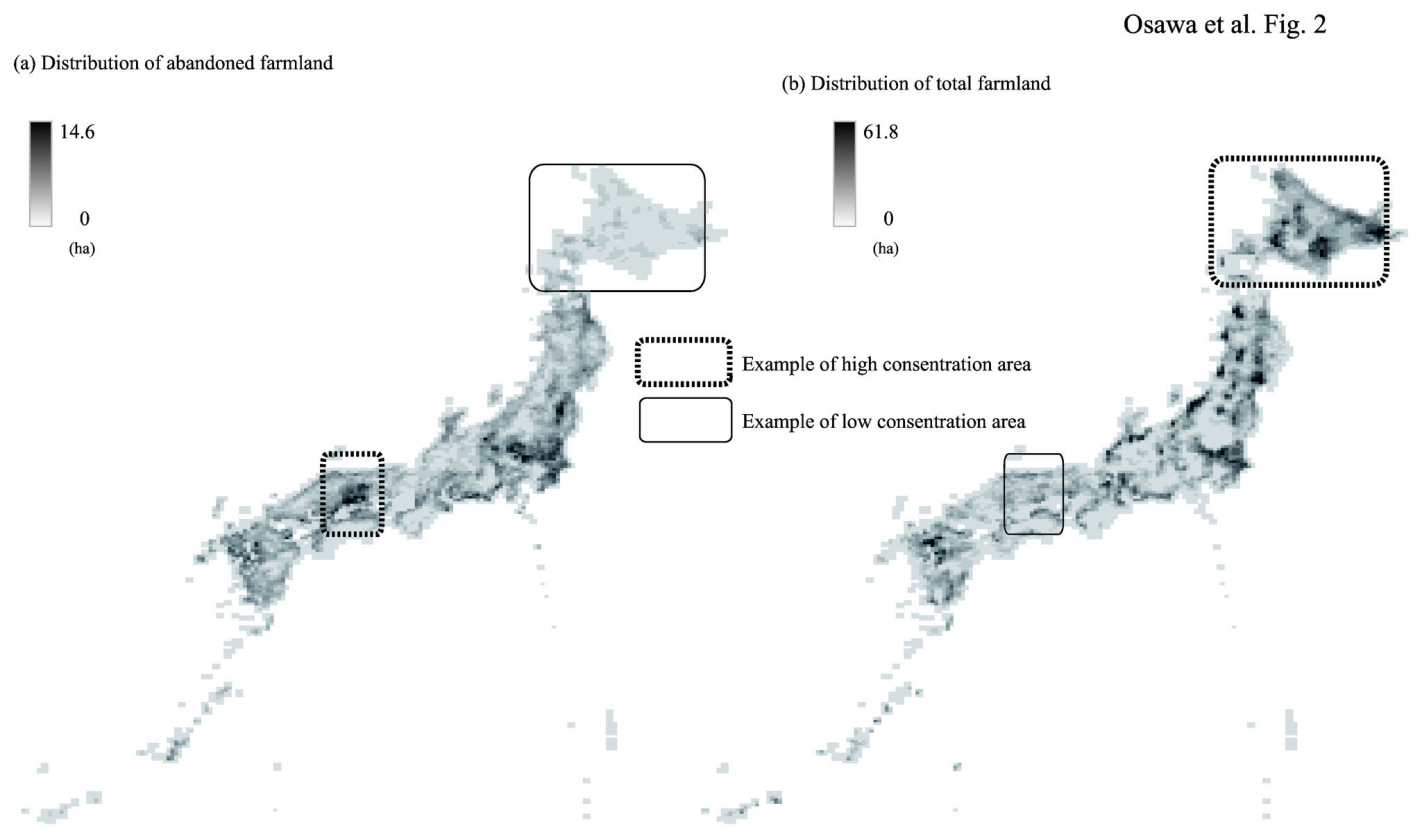
Distribution maps of abandoned farmland (a) and total farmland (b).

Distribution maps for both abandoned farmland areas (a) and total farmland (b) in Japan are presented in Figure 2. The distribution trends appear to differ between these land-use types because high concentration areas differed between abandoned farmland and total farmland (Figure 2).

The average abandoned area per 2nd mesh unit across the whole of Japan was 80.62 ± 115.39 (mean ± SD) ha and was 87.88 ± 117.71 ha when meshes without abandoned area were excluded (Table 2). In both years (2001 and 2007), the average abandoned area per 2nd mesh unit within the ranges of all 23 PT species significantly exceeded the average abandoned area per 2nd mesh unit when the meshes without abandoned land were excluded (Table 2).

The GLM analyses indicated that for 18 PT species, occurrences were positively influenced by the area of abandonment in both years (Table 3; detailed results are presented in Table S2). The other four species were not influenced by the area of abandonment in either year (Table 3). One species (*Gnaphalium hypoleucum*) showed differing results between 2001 and 2007 (Table 3). 

**Table 3:** Results of GLMs based on Wald test for the presence/absence (non record) of each PT species.

	2001		2007	
Species name	Abandoned area	Total farmland	Abandoned area	Total farmland
*Vincetoxicum pycnostelma*	***	n.s.	***	n.s.
*Penthorum chinense*	***	***	***	***
*Utricularia uliginosa*	n.s.	***	n.s.	***
*Isoetes japonica*	***	***	***	***
*Salvinia natans*	***	***	***	***
*Leonurus macranthus*	**	n.s.	**	n.s.
*Potamogeton berchtoldii*	**	***	***	***
*Azolla japonica*	***	**	***	***
*Gnaphalium hypoleucum*	***	**	*	n.s.
*Sparganium stenophyllum*	*	n.s.	*	***
*Marsilea quadrifolia*	***	*	***	**
*Saussurea pulchella*	*	n.s.	*	n.s.
*Sparganium fallax*	*	**	**	***
*Monochoria korsakowii*	**	***	*	***
*Blyxa echinosperma*	**	n.s.	***	n.s.
*Mosla japonica*	***	n.s.	***	n.s.
*Trapa incisa*	n.s.	***	n.s.	***
*Ixeris chinensis subsp. strigosa*	***	n.s.	***	n.s.
*Nymphoides peltata*	***	***	***	***
*Persicaria taquetii*	n.s.	***	n.s.	***
*Caldesia parnassifolia*	n.s.	***	n.s.	***
*Persicaria foliosa var. paludicola*	***	*	***	*
*Najas gracillima*	**	n.s.	***	*

Statistical significance are shown. All significant factors were positively influenced for precenses of PT species. Estimated values were shown at appendix table 2. Significant effects identified by Wald tests are provided (n.s.: not significant, * p<0.05, ** p<0.01, *** p<0.001)

Fourteen PT species occurrences were positively influenced by the total farmland area in both years (Table 3). Seven species were not influenced by the total farmland area in either year (Table 3). The other two species showed differing results between 2001 and 2007 (Table 3).

Positive or negative effects of abandoned farmland area and total farmland area for individual PT species, as identified in the bagging GLM results, are shown in Table 4. For all 23 PT species, occurrences were positively influenced by the area of abandonment in both years (Table 4; detailed results are shown in Table S3). For 20 PT species, occurrences were positively influenced by total farmland area in both years (Table 4). Occurrences of three PT species, *Leonurus macranthus*, *Saussurea pulchella* and *Mosla japonica*, were negatively influenced by total farmland area in both years (Table 4). Significant effects on these three species were not detected in the GLM results (Table 3).

**Table 4:** Results of Bagging GLMs for the presence/absence (no-record) of each PT species.

	2001		2007	
Species name	Abandoned area	Total farmland	Abandoned area	Total farmland
*Vincetoxicum pycnostelma*	+	+	+	+
*Penthorum chinense*	+	+	+	+
*Utricularia uliginosa*	+	+	+	+
*Isoetes japonica*	+	+	+	+
*Salvinia natans*	+	+	+	+
*Leonurus macranthus*	+	-	+	-
*Potamogeton berchtoldii*	+	+	+	+
*Azolla japonica*	+	+	+	+
*Gnaphalium hypoleucum*	+	+	+	+
*Sparganium stenophyllum*	+	+	+	+
*Marsilea quadrifolia*	+	+	+	+
*Saussurea pulchella*	+	-	+	-
*Sparganium fallax*	+	+	+	+
*Monochoria korsakowii*	+	+	+	+
*Blyxa echinosperma*	+	+	+	+
*Mosla japonica*	+	-	+	-
*Trapa incisa*	+	+	+	+
*Ixeris chinensis subsp. strigosa*	+	+	+	+
*Nymphoides peltata*	+	+	+	+
*Persicaria taquetii*	+	+	+	+
*Caldesia parnassifolia*	+	+	+	+
*Persicaria foliosa var. paludicola*	+	+	+	+
*Najas gracillima*	+	+	+	+

All estimated values were shown at appendix table 3. Contributions of positive (+) or negative (-) are shown. "+" means that variables were positively influenced for the species occurrences. "-" means that variables were negatively influenced for the species occurrences.

## Discussion

We compared the average abandoned areas in 2nd mesh units within 23 PT species distribution ranges to the average for all of Japan to test the relationship between agricultural abandonment and PT species distributions. In all cases, the average area of abandoned farmland per mesh unit was larger within PT species ranges than across the whole of the country in both years tested. Also, 18 species occurrences were positively influenced by abandoned areas despite the patchy analysis. Additionally, all 23 PT species were positively influenced by abandoned areas in the machine learning analysis results. Therefore, a relationship exists between the spatial patterns of abandonment and the distributions of threatened plant species that should be incorporated into planning for the management of biodiversity in land tracts undergoing shifts in human usage.

### Dependency of PT species on farmland

Most of the PT species grew in agricultural habitats such as paddy fields, ponds and ditches (Table 1). The occurrences of 14 species were positively influenced by the total area of farmland, and all species studied were influenced by either abandoned area or total farmland area (GLM analysis; Table 3). In addition, the occurrences of 20 PT species were positively influenced by the total area of farmland (bagging GLM results; Table 4). These results suggest that nearly all PT species are likely to depend on farmland, especially paddy fields. Paddy fields represent a major portion of agricultural land use in Japan. Therefore, our expectation of which PT species depend on farmland for habitat is generally supported.

### Farmland likely to be abandoned

We demonstrated unequivocally that areas of increasing agricultural abandonment overlap areas where PT species occur in Japan. Also, at least 18 species occurrences were positively influenced by abandoned area in the results of both the GLM and bagging GLM analyses (Tables 3, 4). From a study of four threatened plants, one of which (*V*. *Pycnostelma*) was included in our study, Uematsu et al. [12] showed that threatened plant species are likely to be distributed in paddy fields with a high risk of abandonment. We provide supporting evidence for this idea across the entire Japanese archipelago, thus supporting the generality of this result. This suggests that relationships may exist between farmland likely to be abandoned and habitat for threatened plant species.

### Effects of abandonment on PT species

Our results showed an overlap between abandoned areas and the occurrences of PT species. Many previous studies have suggested that agricultural abandonment negatively affects regional biodiversity in several parts of Japan [10,12,19,21,22,38]. Our findings provide supporting evidence and offer a panoramic view of biodiversity losses over large agricultural landscapes. Several mechanisms that drive biodiversity loss when farmlands are abandoned have been proposed in the past. These mechanisms include reduced rates of disturbance [22] and changes in water conditions after abandonment [12,38]. Thus, biodiversity losses are likely driven by multiple factors after land-use shifts. In fact, our 23 PT species included several life-forms, morphologies and diverse habitat requirements (Table 1); declining numbers of such species are highly unlikely to be attributable to a single mechanism. Thus, agricultural abandonment probably affects plant species with diverse ecological traits through multiple mechanisms [12]. Water is likely a key component of the mechanisms because the three habitats most occupied by the 23 PT species were paddies, ponds and ditches (Table 1); furthermore, previous research has shown that water dynamics in agricultural fields strongly influence several plant species [23]. Future investigations should examine in more detail the processes of agricultural abandonment that cause population declines for each species and/or species trait clusters because the influences of agricultural abandonment are determined by species-specific habitat preferences and/or ecological traits [12]. 

Our results also suggest that abandoned farmlands provide habitats for some PT species. Ikegami et al. [23] indicated that agricultural abandonment may recover natural habitat from human-managed environments. From this viewpoint, habitat recovery should currently be progressing through all of Japan, with positive influences on regional biodiversity. In fact, agricultural abandonment has occurred relatively recently, since 1990 [11,12], whereas biodiversity losses generally occurred before 1970 [39]. Thus, some of the PT species occurrences might have been the result of habitat recovery following agricultural abandonment. Nevertheless, many previous studies have suggested that agricultural abandonment negatively affects regional biodiversity. Conditions might exist under which abandonment has different effects on PT species.

### Differences within abandoned areas

Our results suggest that agricultural abandonment has contradictory effects on plant diversity compared to previous conclusions. Here, we propose two interpretations for both positive and negative effects of agricultural abandonment that explain our results and those of previous studies. The first is the potential natural habitat effect. As agricultural enterprise changes natural habitats to human-managed habitats [1,2], the former native habitats might have been more diverse originally. For example, before conversion to single-use agriculture, a given habitat may have supported wetlands, forests and grasslands. Agricultural abandonment might recover some these natural habitats, which may or may not be suitable for PT species. Thus, the influence of abandonment can be positive or negative depending on the suitability of habitats that emerge from the process. 

Our second interpretation involves land-use history effects. Specifically, consolidation work, such as heavy land levelling, in farmland might negatively influence habitat conditions for plant species. Ikegami et al. [23] indicated that abandoned farmlands harbour some threatened species under existing conditions, i.e. with no consolidation work. Consolidation work may negatively influence plant species diversity over protracted time periods [12,23]. Recently, agriculture developments have been consolidated in high-yield areas for economic reasons [39,40]. Furthermore, technological developments for agriculture provide increasing yields per unit area. As a result, we can expect increases in the areas of previously consolidated but subsequently abandoned farmlands, and this process may inevitably have negative effects on plant species diversity. With our data, we were not able to demonstrate which of these positive and negative effects is more likely in chosen sections of landscape because we used a macro-scale approach that ignored local fine-scale details [41,42,43]. Therefore, future investigations should also examine fine-scale details of original habitat (pre-agriculture) and land-use history (experience of consolidation work) within regions of increasing farmland abandonment.

## Conclusions

We showed that areas of increasing farmland abandonment overlap agricultural sectors harbouring PT species. These results suggest that abandonment might have positive or negative influences on the survival of several plants distributed across the whole of Japan. In several regions of the planet in addition to Japan, agricultural areas that are less productive and less accessible due to steep topography and poor road conditions are more likely to be abandoned [12,14,44,45]. Biodiversity conservation efforts should focus on such landscapes because increasing agricultural abandonment is an important factor for the loss or conservation of species richness in agricultural ecosystems. Agricultural ecosystems are altered by human activities [1,2]. Thus, despite the fact that active farmland and abandoned areas have similar surface appearances, several intrinsic factors may exist that influence biodiversity, such as the availability of natural habitat and previous land use. From the perspective of biodiversity conservation, these intrinsic factors are very important. To conserve biodiversity in agricultural ecosystems, policy makers should pay more attention to the management of agricultural areas, especially abandoned areas, giving due consideration to the potential natural habitats and historical effects of agricultural land use.

## Supporting Information

Figure S1 **Total agricultural abandonment areas in Japan from 1990 to 2010.**
(EPS)

Table S1 **Published years on current PRDBs.**
(DOCX)

Table S2 **Results of GLMs for the presence/absence of each PT species.** Estimated coefficients and standard deviations are shown.
(DOCX)

Table S3 **Results of Bagging GLMs for the presence/absence of each PT species.** Estimated coefficients and standard deviations are shown. The average values of the coefficients of the 90% confidence interval of all 5,000 models were used.
(DOCX)
