# Supplementary material for: Areas of Increasing Agricultural Abandonment Overlap the Distribution of Previously Common, Currently Threatened Plant Species
Source: PLoS One. 2013 Nov 8;8(11):e79978. doi: 10.1371/journal.pone.0079978 (PMC3832657; doi:10.1371/journal.pone.0079978)
Supplement: Supplementary file 1 [file pone.0079978.s002.docx]

Table S1. Published years on current PRDBs.

Prefecture	Published year
