# Supplementary material for: Areas of Increasing Agricultural Abandonment Overlap the Distribution of Previously Common, Currently Threatened Plant Species
Source: PLoS One. 2013 Nov 8;8(11):e79978. doi: 10.1371/journal.pone.0079978 (PMC3832657; doi:10.1371/journal.pone.0079978)
Supplement: Supplementary file 2 [file pone.0079978.s003.docx]

Table S2. Results of GLMs for the presence/absence of each PT species. Estimated coefficients and standard deviations are shown.

	2001						2007			
Species name	Intercept	se	Abandoned	se	Total farmland	se	Intercept	se	Abandoned	se
Vincetoxicum pycnostelma	-2.85100	0.09438	0.00182	0.00045	0.00009	0.00007	-2.58982	0.08294	0.00249	0.00039
Penthorum chinense	-3.63900	0.11710	0.00346	0.00042	0.00044	0.00006	-3.45600	0.10720	0.00345	0.00039
Utricularia uliginosa	-3.12000	0.10700	0.00068	0.00055	0.00026	0.00007	-2.89800	0.09760	0.00058	0.00052
Isoetes japonica	-3.65600	0.12180	0.00259	0.00045	0.00041	0.00006	-3.26700	0.10340	0.00248	0.00041
Salvinia natans	-3.93000	0.13490	0.00295	0.00046	0.00044	0.00007	-3.43500	0.10970	0.00257	0.00042
Leonurus macranthus	-3.30883	0.12212	0.00164	0.00061	-0.00013	0.00011	-3.08913	0.11091	0.00161	0.00057
Potamogeton berchtoldii	-3.88561	0.14290	0.00182	0.00058	0.00031	0.00008	-3.44600	0.11550	0.00197	0.00047
Azolla japonica	-4.24000	0.15700	0.00380	0.00051	0.00026	0.00009	-3.99500	0.13940	0.00352	0.00047
Gnaphalium hypoleucum	-3.92700	0.14510	0.00289	0.00054	0.00014	0.00010	-3.46400	0.12790	0.00127	0.00064
Sparganium stenophyllum	-3.90141	0.15029	0.00156	0.00067	0.00018	0.00010	-3.50200	0.12340	0.00124	0.00057
Marsilea quadrifolia	-4.25000	0.16000	0.00366	0.00052	0.00022	0.00009	-3.89300	0.13650	0.00351	0.00048
Saussurea pulchella	-3.56833	0.14041	0.00156	0.00072	-0.00020	0.00014	-3.38361	0.12987	0.00141	0.00069
Sparganium fallax	-4.05700	0.15720	0.00166	0.00066	0.00028	0.00009	-3.66300	0.13170	0.00152	0.00058
Monochoria korsakowii	-4.92800	0.20670	0.00174	0.00068	0.00076	0.00008	-4.47500	0.17170	0.00145	0.00062
Blyxa echinosperma	-4.11439	0.16391	0.00197	0.00068	0.00016	0.00011	-3.87800	0.14350	0.00263	0.00055
Mosla japonica	-4.03200	0.16270	0.00235	0.00068	-0.00004	0.00013	-3.92800	0.15260	0.00250	0.00062
Trapa incisa	-4.60600	0.19360	0.00129	0.00076	0.00054	0.00009	-4.53700	0.18650	0.00097	0.00075
Ixeris chinensis subsp. strigosa	-4.14100	0.17130	0.00257	0.00069	-0.00009	0.00015	-3.93400	0.15230	0.00233	0.00062
Nymphoides peltata	-5.23300	0.23290	0.00245	0.00067	0.00072	0.00008	-4.77900	0.19260	0.00230	0.00060
Persicaria taquetii	-4.66900	0.20370	0.00142	0.00082	0.00044	0.00010	-4.37500	0.17820	0.00113	0.00075
