# Supplementary material for: Areas of Increasing Agricultural Abandonment Overlap the Distribution of Previously Common, Currently Threatened Plant Species
Source: PLoS One. 2013 Nov 8;8(11):e79978. doi: 10.1371/journal.pone.0079978 (PMC3832657; doi:10.1371/journal.pone.0079978)
Supplement: Supplementary file 3 [file pone.0079978.s004.docx]

Table S3. Results of Bagging GLMs for the presence/absence of each PT species. Estimated coefficients and standard deviations are shown.
The average values of the coefficients of the 90% confidence interval of all 5,000 models were used.

	2001						2007				
Species name	Intercept	se	Abandoned	se	Total farmland	se	Intercept	se	Abandoned	se	Total farmland
Vincetoxicum pycnostelma	-0.47	0.0011	0.0028	0.000009	0.00017	0.0000012	-0.47	0.0011	0.0028	0.000009	0.00017
Penthorum chinense	-1.18	0.0013	0.0043	0.000011	0.00059	0.0000013	-1.18	0.0013	0.0043	0.000011	0.00059
Utricularia uliginosa	-0.37	0.0013	0.0009	0.000011	0.00034	0.0000016	-0.37	0.0013	0.0009	0.000011	0.00034
Isoetes japonica	-0.90	0.0013	0.0027	0.000012	0.00058	0.0000020	-0.90	0.0013	0.0027	0.000012	0.00058
Salvinia natans	-1.00	0.0014	0.0031	0.000012	0.00062	0.0000019	-1.00	0.0014	0.0031	0.000012	0.00062
Leonurus macranthus	-0.15	0.0017	0.0025	0.000017	-0.00017	0.0000017	-0.15	0.0017	0.0025	0.000017	-0.00017
Potamogeton berchtoldii	-0.77	0.0017	0.0026	0.000016	0.00050	0.0000024	-0.77	0.0017	0.0026	0.000016	0.00050
Azolla japonica	-1.04	0.0018	0.0046	0.000015	0.00040	0.0000017	-1.04	0.0018	0.0046	0.000015	0.00040
Gnaphalium hypoleucum	-0.23	0.0015	0.0017	0.000015	0.00007	0.0000019	-0.23	0.0015	0.0017	0.000015	0.00007
Sparganium stenophyllum	-0.55	0.0017	0.0012	0.000013	0.00048	0.0000026	-0.55	0.0017	0.0012	0.000013	0.00048
Marsilea quadrifolia	-0.88	0.0017	0.0041	0.000014	0.00033	0.0000018	-0.88	0.0017	0.0041	0.000014	0.00033
Saussurea pulchella	-0.07	0.0019	0.0021	0.000019	-0.00023	0.0000020	-0.07	0.0019	0.0021	0.000019	-0.00023
Sparganium fallax	-0.60	0.0020	0.0020	0.000016	0.00043	0.0000025	-0.60	0.0020	0.0020	0.000016	0.00043
Monochoria korsakowii	-1.17	0.0017	0.0013	0.000015	0.00088	0.0000025	-1.17	0.0017	0.0013	0.000015	0.00088
Blyxa echinosperma	-0.72	0.0025	0.0042	0.000022	0.00023	0.0000026	-0.72	0.0025	0.0042	0.000022	0.00023
Mosla japonica	-0.46	0.0023	0.0043	0.000026	-0.00008	0.0000025	-0.46	0.0023	0.0043	0.000026	-0.00008
Trapa incisa	-1.13	0.0027	0.0015	0.000020	0.00090	0.0000035	-1.13	0.0027	0.0015	0.000020	0.00090
Ixeris chinensis subsp. strigosa	-0.51	0.0024	0.0036	0.000022	0.00010	0.0000025	-0.51	0.0024	0.0036	0.000022	0.00010
Nymphoides peltata	-1.32	0.0023	0.0020	0.000012	0.00092	0.0000032	-1.32	0.0023	0.0020	0.000012	0.00092
Persicaria taquetii	-0.74	0.0020	0.0010	0.000015	0.00064	0.0000029	-0.74	0.0020	0.0010	0.000015	0.00064
Caldesia parnassifolia	-1.07	0.0027	0.0010	0.000020	0.00092	0.0000037	-1.07	0.0027	0.0010	0.000020	0.00092
